# Critical Role of Aberrant Angiogenesis in the Development of Tumor Hypoxia and Associated Radioresistance

**DOI:** 10.3390/cancers6020813

**Published:** 2014-04-08

**Authors:** Gabriele Multhoff, Jürgen Radons, Peter Vaupel

**Affiliations:** 1 Department of Radiotherapy and Radiation Oncology, Klinikum rechts der Isar, Technische Universität München, Ismaninger Straße 22, 81675 Munich, Germany; E-Mail: peter.vaupel@lrz.tum.de; 2 Clinical Cooperation Group “Innate Immunity in Tumor Biology”, Helmholtz Zentrum München (HMGU), Ingolstädter Landstraße 1, 85764 Neuherberg, Germany; 3 multimmune GmbH, Munich, Ismaningerstr. 22, 81675 Munich, Germany; E-Mail: raj10062@web.de

**Keywords:** tumor angiogenesis, tumor microcirculation, tumor hypoxia, hypoxia-inducible factor (HIF), radioresistance, hypoxia modifiers

## Abstract

Newly formed microvessels in most solid tumors show an abnormal morphology and thus do not fulfil the metabolic demands of the growing tumor mass. Due to the chaotic and heterogeneous tumor microcirculation, a hostile tumor microenvironment develops, that is characterized *inter alia* by local hypoxia, which in turn can stimulate the HIF-system. The latter can lead to tumor progression and may be involved in hypoxia-mediated radioresistance of tumor cells. Herein, cellular and molecular mechanisms in tumor angiogenesis are discussed that, among others, might impact hypoxia-related radioresistance.

## 1. Introduction

Endothelial cells build up the first “barrier” between the blood, interstitial space (and stroma) and parenchymal cells. A dense network of blood vessels is necessary to provide an adequate supply of oxygen and nutrients, and efficient drainage of waste products. Although the turnover rate of endothelial cells is generally slow in adult organs, endothelial cell growth can be induced under (patho-)physiological conditions like wound healing, menstrual cycle or placenta formation. As shown for normal tissues also growth of solid tumors depends on blood vessels. Vasculogenesis, arteriogenesis and angiogenesis are three major principles to build up new vessels. Vasculogenesis is a process that involves undifferentiated progenitor cells in order to form a vascular network. Vasculogenesis is required for the *de novo* formation of a vascular network in embryogenesis and growth [1]. In contrast to vasculogenesis, arteriogenesis refers to the remodelling of pre-existing arterioles to form arteries upon, e.g., increased shear stress. Arteriogenesis is based on chemokine/growth factor-induced growth processes and enlargement of vascular wall structures at larger shear stress that is induced by increased blood flow rates in arteries [2]. During angiogenesis vessels are formed from the existing microvasculature. The mechanism of angiogenesis involves either sprouting from pre-existing vessels or splitting through intussusception [3,4]. Apart from the female reproductive organs, during pregnancy and in wound healing [5,6], the vasculature rarely forms new branches in adults. However, endothelial cells retain their plasticity to sense and to respond to angiogenic signals during their whole life-time. In general, angiogenesis is tightly regulated by a fine balance of activating and inhibiting signals. Cytokines, hormones, circulating progenitor cells, whose role is not completely understood, endothelial cell migration and destabilization of the vessel wall, the basal lamina, and the interstitial matrix can impact on angiogenesis. Apart from physiological parameters, microenvironmental factors such as hypoxia and nutrient deficiencies can also trigger the angiogenic switch. Angiogenesis is also a crucial player in the pathogenesis of autoreactive diseases such as age-related macular degeneration, rheumatoid diseases, inflammation, arteriosclerosis, vascular restenosis and different vasculopathies. A close link of inflammation and angiogenesis is indicated by hallmark factors of acute and chronic inflammation such as VEGF-A and angiopoietins [7].

## 2. Vessel Formation in Malignant Tumors

Tumor angiogenesis involves the production and release of growth factors, permeability regulating factors, migration stimulating factors, proteolytic enzymes, extracellular matrix and adhesion molecules. These factors can be released either by tumor, stromal and/or inflammatory cells that are located within or in close proximity to the tumor. Growth factors of tumor angiogenesis can either involve specific vascular endothelium factors (*i.e.*, vascular endothelial growth factor (VEGF), angiopoietin and ephrin family members), or non-specific factors (*i.e.*, platelet-derived growth factor (PDGF), transforming growth factor-beta (TGF-β), fibroblast growth factor (FGF) or tumor necrosis factor-α (TNF-α)) [8]. In principle, the progression of tumor growth is critically dependent on oxygen and nutrient supply and the drainage of metabolites [9], since diffusion without the involvement of blood vessels allows transport processes only over very short distances of less than 500 µm.

The physiology of tumors is different from that of normal tissues. It is characterized *inter alia* by O_2_ depletion (hypoxia or anoxia), extracellular acidosis, high lactate and adenosine levels, glucose and bicarbonate deprivation, energy impoverishment, significant interstitial fluid flow, interstitial hypertension, and other adverse conditions characterizing the metabolic tumor microenvironment [10,11,12,13,14]. This hostile microenvironment is largely determined by an abnormal tumor microcirculation. When considering the continuous and indiscriminate formation of a vascular network in growing tumors, the following pathogenetic mechanisms can be involved either alone or in combination:

(a) Angiogenesis by endothelial sprouting from pre-existing venules [15,16].
(b) Co-option of existing vessels [17].
(c) Vasculogenesis (*de novo* vessel formation) through incorporation of circulating endothelial precursor cells [17].
(d) Intussusception (splitting of the lumen of a vessel into two).
(e) Formation of pseudo-vascular channels lined by tumor cells rather than endothelial cells (“vascular mimicry”).
(f) Microvessel formation by a subset of bone marrow-derived myeloid cells infiltrating the tumor [17].

**Figure 1:**
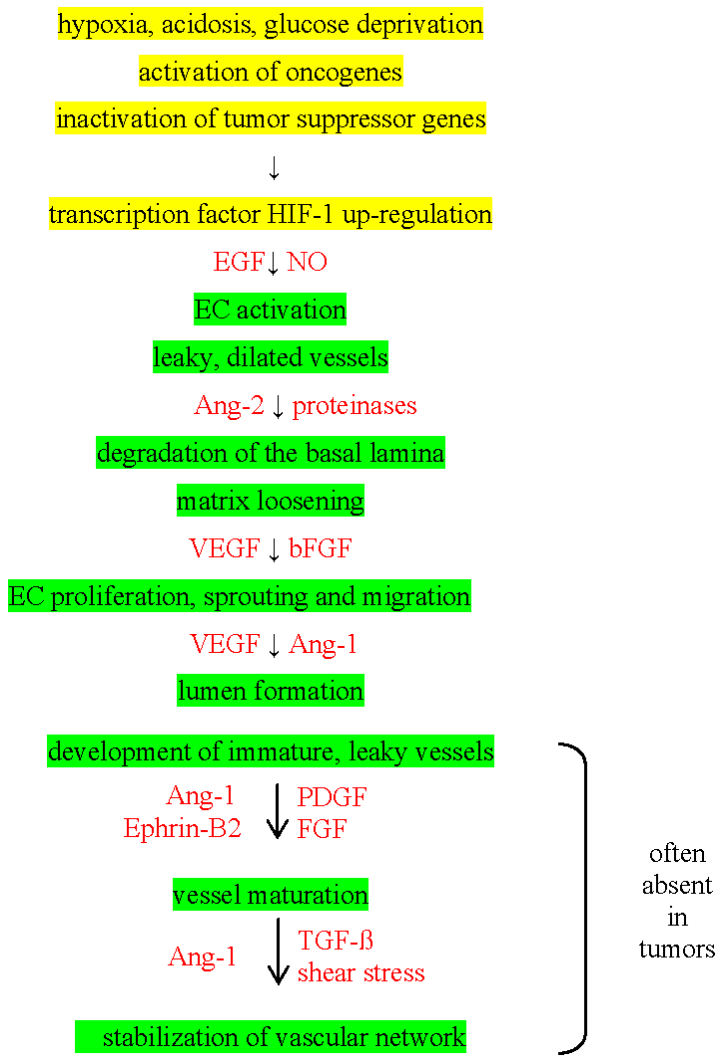
“Chronological” steps in tumor angiogenesis (marked in green). Causative mechanisms are marked in yellow and pro-angiogenic factors are marked in red.

Despite these various possibilities for the formation of tumor microvessels, the tumor vasculature often lacks the signals to mature and therefore, tumor vasculature is also termed “aberrant monster” [18]. Tumor vessels are characterized by vigorous proliferation which leads to immature, structurally defective and, in terms of perfusion, ineffective microvessels (Figure 1). Consequently, tumor blood flow is chaotic and heterogeneous, the vascular supply and the metabolic microenvironment are inadequate and hostile. However, due to the spatio-temporal heterogeneity of pro-angiogenic signals, not all vessels are totally immature in clinical cancers. Some of them actually retain contractile properties [19].

## 3. Angiogenic Switch in Tumors

Small tumors can stay dormant until the so-called angiogenic switch occurs. Neovascularization is driven by pro-angiogenic factors that facilitate the formation of new microvessels from pre-existing blood vessels. This angiogenic switch by which an avascular tumor nodule is converted into a fast growing, vascularized and aggressive tumor is important in tumor growth and dissemination [20].

The angiogenetic switch is regulated by various *pro-angiogenic factors* such as VEGF, IL-8, bFGF, EGF, PDGF, MMP-2/-9, uPA, Notch-1/-4, osteopontin, and angiogenin and *anti-angiogenic factors* such as angiostatin, thrombospondin, IFN-γ, IL-1/-4/-12/-18/-21. Among them, members of the VEGF family (VEGF-A, B, C, D) have been identified as the dominant players in tumorigenesis. VEGF-C has been shown to activate the VEGFR-3 and Notch signalling pathways [21]. Under normoxic conditions, VEGF is mainly regulated by hypoxia-inducible factor 1 (HIF-1) that can be activated by pro-inflammatory cytokines such as IL-1 and TNF via PI3K and NF-κB [22,23], or indirectly by IL-6 and PGE_2_ in an autocrine manner [24,25]. In human tumors the VEGF expression is frequently upregulated [26]. High VEGF levels promote the production of abnormal vessels as mentioned before [27] and binding of VEGF to its corresponding receptor leads to the activation of the PI3K/Akt/mTOR and Ras/Raf/MAPK pathways that in turn promote not only angiogenesis but also proliferation, differentiation and survival of tumor cells [28]. Human osteosarcoma and pancreatic adenocarcinoma cells have been found to spontaneously release high amounts of VEGF, MMP-2, and IL-8 that enhance the invasiveness of tumors [29,30,31]. Via an auto-regulatory loop, the pro-inflammatory cytokine IL-1 further up-regulates the secretion of the pro-angiogenic factor IL-8 and thus supports the growth of tumors [30]. In SW1353 chondrosarcoma cells IL-1 induces a massive release of the pro-angiogenic factors MMP-1 and MMP-13. These findings highlight the crucial impact of inflammatory mediators in tumor angiogenesis [32]. Epidermal growth factor receptor (EGFR) that is also frequently overexpressed in tumors is associated with poor prognosis, high resistance to radiochemotherapy and increased metastatic spread [32]. EGFR predominantly induces the Ras/Raf/MAPK and the PI3K/Akt pathway that are both responsible for anti-apoptotic and pro-survival signals. Pro-angiogenic factors orchestrating the complex angiogenesis in solid tumors also include metabolites/catabolites such as lactate, 3-hydroxybutyrate, succinate and fumarate [33]. The role of irradiation in angiogenesis is still a matter of debate. Whereas some groups speculate that radiation can induce angiogenesis others report on a repression of angiogenesis by ionizing irradiation [34,35,36,37].

## 4. Tumor Microcirculation

As already mentioned, newly formed microvessels in most solid tumors do not conform to the normal morphology of the host tissue vasculature. The tumor vasculature can be described as a system that is maximally stimulated, yet only minimally fulfils the metabolic demands of the growing tumor that it supplies.

Microvessels in solid tumors exhibit a large series of severe structural and functional abnormalities. They are often dilated, tortuous, elongated, and saccular. It is of note that not only the quantity of microvessels counts, but also—or even more so—the quality of vascular function in terms of the tumor tissue supply or drainage [37,38]. There is significant arterio-venous shunt perfusion accompanied by a chaotic vascular organization that lacks any regulation matched to the metabolic demands or functional status of the tissue [11]. Excessive branching is a common finding, often coinciding with blind vascular endings. Incomplete or even missing endothelial lining and interrupted basement membranes result in an increased vascular permeability with extravasation of blood plasma and/or red blood cells expanding the interstitial fluid space and drastically increasing the hydrostatic pressure in the tumor interstitium (interstitial fluid pressure).

In solid tumors, there is a rise in viscous resistance to flow mainly caused by the hemoconcentration (hematocrit increase ranging from 5 to 14%) [39,40]. Aberrant vascular morphology and a decrease in vessel density are responsible for an increase in geometric resistance to flow, which can lead to an inadequate perfusion. Substantial spatial heterogeneity in the distribution of tumor vessels and significant temporal heterogeneity in the microcirculation within a tumor [41,42,43,44] may result in a considerably anisotropic distribution of tumor tissue oxygenation and a number of other factors, which are usually closely linked and which define the so-called pathophysiological microenvironment. Variations in these relevant parameters in different tumors are often more pronounced than differences occurring between different locations or microareas within a tumor [12,45,46].

## 5. Tumor Blood Flow Rates

A number of studies on blood flow through human tumors have been reported. Some of them are anecdotal reports rather than systematic investigations, and therefore, definite conclusions cannot be drawn partly due to the use of non-validated techniques to measure flow in volume flow rate units. Considering the presently available data, the following conclusions can be drawn when flow data derived from different reports are pooled (for reviews see [11,14,17,47]):

(a) Blood flow can vary considerably despite similar histological classification and primary site (0.01–2.9 mL/g/min; [17,48,49].
(b) Tumors can have flow rates which are similar to those measured in organs with a high metabolic rate such as liver, heart or brain.
(c) Some tumors exhibit flow rates which are even lower than those of tissues with a low metabolic rate such as skin, resting muscle or adipose tissue.
(d) Blood flow in human tumors can be higher or lower than that of the tissue of origin, depending on the functional state of the latter tissue (e.g., average blood flow in breast cancers is substantially higher than that of postmenopausal breast and significantly lower than flow data obtained in the lactating, parenchymal breast).
(e) The average perfusion rate of carcinomas does not deviate substantially from that of tissue sarcomas.
(f) Metastatic lesions exhibit a blood supply which is comparable to that of the primary tumor [11].
(g) In some tumor entities, blood flow in the periphery is distinctly higher than in the center whereas in others, blood flow is significantly higher at the tumor center compared to the tumor edge.
(h) Flow data from multiple sites of measurement show marked heterogeneity within individual tumors. In cervical cancer, the intra-tumor heterogeneity was similar to the inter-tumor heterogeneity [50].
(i) There is substantial temporal flow heterogeneity on a microscopic level within human tumors as shown by multichannel laser Doppler flowmetry [51,52].
(j) There is no association between tumor size and blood flow in many cancers [48,53].
(k) Tumor blood flow is not regulated according to the metabolic demand as is the case in normal tissues.

With regard to the efficacy of radiotherapy the effectiveness of blood flow greatly influences the oxygen supply of tumors. Therefore, the responsiveness of solid tumors to radiotherapy (and chemotherapy) profoundly depends on blood perfusion [54].

## 6. Arterio-Venous Shunt Perfusion in Tumors

First rough estimations concerning the arterio-venous shunt flow in malignant tumors showed that at least 30% of the arterial blood can pass through experimental tumors without participating in the microcirculatory exchange processes [55,56,57]. In patients receiving intra-arterial chemotherapy for head and neck cancer, shunt flow is reported to be 8% to 43% of total tumor blood flow, the latter consistently exceeding normal tissue perfusion on the scalp [58]. The mean fractional shunt perfusion of tumors was 23% ± 13% in studies utilizing ^99m^Tc-labeled micro-aggregated albumin (diameter of the particles, 15–90 µm). The significance of this shunt flow on local, intra-tumoral pharmacokinetics, on the development of hypoxia, and on other relevant metabolic phenomena has not yet been systematically studied and remains speculative.

High amounts of shunt flow through solid tumors not only impact on pharmacokinetics of anti-cancer agents, but also limit the effectiveness of radiotherapy due to the development of diffusion-limited, chronic hypoxia [44].

## 7. Tumor Hypoxia and HIF

Aberrant microcirculation is a major causative factor for the development of hypoxia in solid tumors [59]. Hypoxia is strongly associated with radio-resistance of malignant tumors, tumor recurrence after radiation therapy, and poor prognosis in patients subjected to radiation therapy [50,60]. On the one hand, free radicals that are produced by radiation, either directly or indirectly from an interaction with other molecules such as water, can react with H^+^ in the absence of oxygen and thus the target can be chemically restored to its original form. On the other hand, hypoxia can stimulate the HIF system which in turn can lead to tumor progression. It is hypothesized that the heterodimeric transcription factor HIF-1 is also involved in hypoxia-mediated radioresistance of tumor cells [61,62]. However, *in vitro* data from our group indicate that high HIF-1α levels in lung cancer cell lines are not associated with radioresistance [63].

Apart from its role in the development of radioresistance, HIF-1α is crucially involved in tumor angiogenesis, invasion, survival, and growth [64]. Harada *et al.* have demonstrated that irradiation causes an up-regulation of intra-tumoral HIF-1α protein and activity in regions of radiation-induced re-oxygenation of the solid tumor via the PI3K/Akt/mTOR pathway which is responsible for synthesis, stabilization and accumulation of HIF-1α, the oxygen-regulated subunit of HIF-1 [62]. From these results it can be concluded that Akt/mTOR-dependent translation of HIF-1α plays a critical role in the post-irradiation up-regulation of intra-tumoral HIF-1 activity in response to radiation-induced alterations of oxygen availability in solid tumors. Stability of HIF-1α can also be regulated in an oxygen-independent manner by RACK-1 (receptor of activated protein kinase C) through competition with Hsp90 and recruitment of the elongin-C/B ubiquitin ligase complex [65]. As stated by Yoshimura *et al.*, the transactivational activity of HIF-1 is critically regulated by the MAPK/ERK pathway [66]. HIF-1 transactivational activity was found as being suppressed by FIH-1 (factor inhibiting HIF-1) under normoxic conditions via HIF-1α hydroxylation and concomitant blockage of adapter molecule binding [67]. Furthermore, the irradiation-induced HIF-1 activation can also depend on the availability of nitric oxide [68].

## 8. Therapeutic Interventions to Overcome Hypoxia-Related Radioresistance

Since hypoxia is known to protect tumor cells from standard radiation therapy, several strategies have been developed to interfere with hypoxia-related radioresistance of solid tumors [66]. Hyperbaric oxygen therapy, carbogen, nicotinamide and other “flow modifiers” as well as modification of the hemoglobin-O_2_ affinity have been tested to facilitate oxygen delivery to hypoxic regions. Nitroimidazole derivatives such as misonidazole and nimorazole have been used to sensitize tumors to radiation by mimicking the effect of oxygen. Hypoxic cytotoxins (tirapazamine and analogues) are meant to directly kill tumor cells by hydroxyl radicals or oxidizing radicals. Combined treatment strategies consisting of tirapazamine analogues and HIF-1 inhibitors, such as YC-1, have been tested to increase the radio-responsiveness of tumors [69,70,71,72,73]. However, due to the chaotic vascularization in most solid tumors none of these approaches could significantly improve the sensitivity towards ionizing irradiation.

Anti-angiogenesis is another approach that may affect the radiosensitivity of tumors. The key angiogenic factor VEGF is facilitating tumor growth and survival, and therefore most anti-angiogenic strategies aim to interrupt the VEGF pathway. This idea has led to the development of several anti-VEGF reagents including anti-VEGF antibody bevacizumab, anti-VEGF receptor antibody ramucirumab and VEGF antagonist aflibercept. Despite some promising results in clinical trials, the blockade of VEGF signalling also exerts adverse effects such as resistance to VEGF inhibitors as well as hemorrhagic and thrombotic events due to the damage of healthy vessels [71]. In molecular biology a small molecule is defined as a reagent with a low molecular weight of approximately less than 900 Da. These molecules harbour the capacity to rapidly diffuse across cell membranes and thus can enter cells. Small molecule drugs in pharmacology frequently serve as signalling molecules. A wealth of evidence indicates that small-molecule tyrosine kinase inhibitors such as axitinib, brivanib, cediranib, imatinib, motesanib, pazopanib, sorafenib, sunitinib as well as vatalanib and vandetanib harbor promising activity and safety in certain cancer subtypes (for reviews see [66,74,75]).

Attempts to target the tumor microenvironment in order to improve the effects of radiotherapy also comprise the endogenous angiogenesis inhibitors angiostatin [76] and endostatin [77,78,79,80]. Preclinical results of Ke *et al.* demonstrated that the recombinant human endostatin, endostar, can increase the radiation sensitivity of nasopharyngeal carcinomas in a nude mouse model by lowering the VEGF expression [79]. Interestingly, in patients with advanced cervical cancer the combination of endostar with standard chemoradiotherapy was found to improve the early therapy outcome with acceptable adverse effects [79]. Due to the small sample size and the relatively short follow-up period further investigations are needed with respect to long-term effects.

Despite its history as a human teratogen, thalidomide was tested as a putative drug to disrupt tumor angiogenesis. Although thalidomide monotherapy in patients with therapy-resistant uterine carcinomas prolonged the progression-free survival in a phase II trial [81], a phase III trial did not reveal any survival benefit for patients with brain metastases that have been treated with thalidomide in combination with radiotherapy compared to radiotherapy alone [82]. A meta-analysis of eight randomized trials with 2,317 patients with brain tumors confirmed this observation. Whole brain radiotherapy (WBRT) combined with the potential “radiosensitizer” thalidomide did not significantly improve the overall survival, local control and tumor response compared to WBRT alone [83].

Novel approaches in enhancing tumor radiosensitivity include inhibitors of distinct molecular pathways and key signalling factors such as Ras/Raf/MAPK, PI3K/Akt/mTOR (rapalogs, NVP-BEZ235, NVP-BGT226), c-Kit (imatinib, amuvatinib—also known as MP470), EGFR (cetuximab, erlotinib, sunitinib), PDGFR (sunitinib), and Hsp90 (NVP-AUY922). Cetuximab plus radiotherapy significantly improved the 5-year overall survival compared to radiotherapy alone in patients with locoregionally advanced head and neck tumors [84]. Preclinical studies with the multi-tyrosine kinase inhibitor eunitinib indicate that this drug enhances the radiosensitivity of human prostate cancer [85].

Targeting tumor cells with the EGFR inhibitor erlotinib followed by radiation delayed tumor re-growth to a greater extent than radiation alone [85]. The increase in radiosensitivity by erlotinib was accompanied by a down-regulation of HIF-1 and VEGF, decreased vascular permeability, an increase in tumor blood flow, and a decrease in hypoxia. In a phase I trial, the safety and tolerability of therapy with the mTOR inhibitor everolimus in combination with radiation and temozolomide (TMZ) was evaluated in patients with newly diagnosed glioblastoma multiforme (GBM) [86]. As demonstrated in this study, the combination of everolimus with a standard chemoradiotherapy in patients with GBM was reasonably well tolerated. Moreover, early FDG-PET imaging one week after an everolimus monotherapy revealed a partial metabolic response in a subset of the patients. The efficacy of adding the anti-VEGF antibody bevacizumab and everolimus to standard radiation therapy plus TMZ in the first-line therapy of patients with glioblastoma has been shown to be feasible and safe [87]. The progression-free survival was improved compared to standard radiation therapy plus TMZ. These data are in line with results achieved in other phase II trials in which bevacizumab was used as a fist-line therapy [87]. At present, phase III clinical trials are ongoing to clarify the role of bevacizumab in glioblastoma patients.

A novel approach to radiosensitize tumors is the use of the Hsp90 inhibitor NVP-AUY922. This compound was found to radiosensitize cervical, colorectal and head and neck squamous cell carcinoma (HNSCC) cell lines with a greater potency than any other tested Hsp90 inhibitor *in vitro* and *in vivo* [88]. Moreover, NVP-AUY922 in combination with radiotherapy resulted in a delayed growth of human prostate cancer cells in a mouse model in a supra-additive manner [89]. This effect is due to an oxygen-independent degradation of HIF-1α [65]. These data indicate that the interference of signalling pathways related to hypoxia might improve the radiosensitivity of tumors.

## 9. Conclusions

As summarized in this review, the dynamic and complex tumor microenvironment is largely determined by an aberrant tumor microcirculation characterized, among others, by hypoxia leading to radioresistance of malignant tumors and promoting tumor progression via stimulation of HIF-1. However, more detailed information is needed to characterize the dynamic aspects of tumor hypoxia during treatment. A better understanding of signalling pathways related to hypoxia in the tumor microenvironment should help to develop clinical approaches that address radioresistant hypoxic tumors.

## Abbreviations

Akt: protein kinase B (PKB)
Ang: Angiopoietin
EC: endothelial cell
EGF: epidermal growth factor
EGFR: EGF receptor
bFGF: basic fibroblast growth factor
ERK: extracellular signal-regulated kinase
FIH-1: factor inhibiting HIF-1
FDG PET: fluorine-18-fluorodeoxyglucose positron emission tomography
HIF: hypoxia-inducible factor
HSP: heat shock protein
IL: Interleukin
IFN: Interferon
MAPK: mitogen-activated protein kinase
MMP: matrix metalloproteinase
mTOR: mammalian target of rapamycin
NF-κB: nuclear factor kappa-B
PDGF: platelet-derived growth factor
PDGFR: PDGF receptor
PGE_2_: prostaglandin E_2_
PI3K: phosphatidylinositol 3-kinase
RACK-1: receptor of activated protein kinase C
Raf: rapidly growing fibrosarcoma protein
Ras: rat sarcoma protein
TGF-β: transforming growth factor beta
TMZ: temozolomide
TNF: tumor necrosis factor
uPA: urokinase-type plasminogen activator
VEGF: vascular endothelial growth factor
VEGFR: VEGF receptor
